# Comparison of camel tear proteins between summer and winter

**Published:** 2011-02-01

**Authors:** Ziyan Chen, Farrukh A. Shamsi, Kaijun Li, Qiang Huang, Ali A. Al-Rajhi, Imtiaz A. Chaudhry, Kaili Wu

**Affiliations:** 1 Zhongshan Ophthalmic Center, State Key Laboratory of Ophthalmology, Sun Yat-sen University, Guangzhou, China; 2 King Khaled Eye Specialist Hospital, Riyadh 11462, Saudi Arabia; 3 L.J. Memorial Hospital, Yousuf Building, Yousuf Road, Rasalgunj, Aligarh, India

## Abstract

**Purpose:**

Proteins in the tear fluid have positive effects on maintaining the integrity and stabilization of the tear film, which is affected by several environmental factors. The aim of this study is to investigate seasonal variation of protein patterns in camel tears collected during the summer and winter season.

**Methods:**

Tears from both eyes of 50 clinically normal camels (*Camelus dromedarius*) were collected in the summer (June – July) and in the winter (December – January) respectively. Pooled tear protein samples from two seasons were separated by SDS–PAGE and two-dimensional electrophoresis (2-DE). Protein spots of differential expression in two season gels were excised and subjected to in-gel digestion and identification by matrix assisted laser desorption/ionization-time of flight/time of flight-mass spectrum (MALDI-TOF/TOF-MS) analysis. Two differentially expressed proteins, lactoferrin (LF) and vitelline membrane outer layer protein 1 homolog (VMO1 homolog), were validated by western blotting.

**Results:**

Thirteen well resolved bands were detected in SDS–PAGE gels of both summer and winter camel tears. By band densitometry, significantly higher intensities of band 6, 7, 11, and lower intensity of band 13 were observed in the summer group compared to the winter group. In 2-DE profiles of camel tears, four protein spots were found expressed differentially in two seasons. Further protein identification by MALDI-TOF/TOF-MS and confirmation by western blotting indicated that there was a significant decrease in LF (p=0.002) and an increase in VMO1 homolog (p=0.042) in tears in the summer compared to the winter.

**Conclusions:**

The seasonal variation of camel tear fluids has been found in the composition of proteins, including LF and VMO1 homolog. This result will expand our knowledge of physiologic characteristics of tear fluids and establish a foundation for the mechanistic studies and clinical practices on ocular surface disorders.

## Introduction

The tear film is considered to have a unique structure with functions of nourishing, lubricating and protecting the ocular surface, containing lipid, protein, and mucous components [1,2]. Proteins in the tear film are believed to play an important role in defending the ocular surface from the pathogens, maintaining the integrity and stability of the tear film, and modulating the ocular wound healing process [2-4]. In recent studies, multiple proteomics techniques have been used in the analysis of tear proteins, as potential biomarkers for systemic and ocular diseases [5-9]. The comprehensive and comparative analysis of tear proteins can be helpful in the studies of pathophysiological mechanisms and diagnosis of ocular surface diseases.

The healthy ocular surface is associated with normal tear production and the stability of the tear film, which are found to be affected by several endogenous and exogenous factors, such as age, gender, the time of day, and environmental conditions [10,11]. Studies on humans and animals have reported the daily variations of tear production. Webber et al. [12] by fluorophotometric methods demonstrated that human tear turnover rates in the morning (before 1:00 PM) were significantly greater than in the afternoon (after 1:00 PM). Smith et al. [13] using Schirmer tear test 1 revealed a significant diurnal pattern in dog tear production with the lowest level at midday and highest level in the late afternoon/early evening. A circadian rhythm of tear production during the 12h/12h light/dark period and constant darkness has been reported in horses [11]. The potential influence of season has also been evaluated in tear production of normal horses by Schirmer tear test. However, no statistically different Schirmer tear test values were found in winter compared to the respective values in summer [14].

Reviewed data showed that several environmental risk factors (e.g., relative humidity, temperature, air velocity, and air particles) are associated with alteration of the precorneal tear film (PTF), which was involved in an increase in ocular surface tear film evaporation, alterations in tear secretion and the decrease of goblet cell density, and may subsequently exacerbate development of ocular discomfort as eye irritation symptoms [10,15]. The findings of Sunwoo et al. [16] suggested that it is necessary to maintain greater than 30% relative humidity to avoid dryness of the eyes. A 1 °C decrease in room temperature (within 22 °C to 26 °C) was found to be associated with 19% decrease of the mean value for severity of reported eye irritation in an epidemiological crossover study in public office buildings [17]. Studies on the epidemiology of dry eye disease have revealed that multiple environmental factors (e.g., dry weather, windy condition, long hours of sunlight, and ultraviolet radiation) contribute to the higher prevalence of dry eye [18,19]. Also, the development of several types of conjunctivitis was associated with the seasonal/environmental changes. Laaidi [20] revealed that the weather types of windy conditions, low relative humidity, precipitation below 2 mm and temperatures above 6 °C, which assisted pollen dispersal, could act on the development of allergic conjunctivitis symptoms [20]. Exposure to wind, dust, bright light, and hot weather exacerbated intense pruritus symptoms of vernal keratoconjunctivitis, which is recurrent seasonally in the spring and more common in temperate zones such as central and West Africa and the Middle East [21,22]. These reports suggest that the time and environment conditions of tear evaluation are significant in the diagnosis and treatment of ocular surface disorders.

It is known that the camel survives in the extremely harsh desert condition characterized by a long, hot (temperature highs of above 50 °C), dry (near zero relative humidity) and dusty (sand storms lasting for days) summer season and short but extremely cold and rainy (temperatures falling below 0 °C) winter season. Camel eyes and lacrimal apparatus have been studied in detail [23]. The camel's eyes are protected from blowing sand and dust by a double row of eyelashes and three eyelids on each eye. The extra eyelid also helps protect against the blazing sun, and stops them from going blind. Characterization of tear components in camels might provide some insight into the mechanisms of stabilization of tear film under harsh environmental conditions and disease-induced changes in ocular disorders. Gionfriddo et al. [24,25] have analyzed llama tears by SDS–PAGE and western blotting techniques. The homologs of lysozyme, LF, IgA, transferrin, ceruloplasmin, α1-antitrypsin, α1-amylase, α2–macroglobulin, and proteases were detected in llama tears. However, to our knowledge, no studies focused on the evaluation of the seasonal variation of protein profiles in tears by proteomics techniques.

In the current study, we have used two-dimensional electrophoresis (2-DE) and matrix assisted laser desorption/ionization-time of flight/time of flight-mass spectrum (MALDI-TOF/TOF-MS) to make a comparative proteomics analysis of camel tears collected in the summer and winter seasons. This is the first study to examine seasonal variation of proteins in camel tears, as a basis of discovering biochemical characterization of tear proteins to make improvements in diagnosis, treatment and prognosis of ocular surface disorders in humans.

## Methods

### Tear sampling

The protocol for the collection of tear samples was approved by the Institutional Review Board of the King Khaled Eye Specialist Hospital, Riyadh, Saudi Arabia.

Camel owners outside Riyadh, Saudi Arabia were contacted and approached for collecting camel tears. Tears were collected from both eyes of 50 clinically normal camels in the morning controlled in the summer (June – July) and the winter (December - January) separately. All animals had no signs of disease of the external ocular structures. No agents to induce lacrimation or anesthetic were used for the collection of tears. Tears were collected with a 50 μl sterile plastic pipette by placing it in the lower conjunctival fornix. Care was taken to cause as little conjunctival trauma as possible during collection. The animal samples were immediately stored on ice and brought back to the laboratory for further processing. Unless otherwise stated, all the tear samples were centrifuged at 10,000× g for 5 min at 4 °C to remove gross debris and mucus and pooled. The concentration of protein in the sample was measured by the BCA method using BSA (Shanghai Shengzheng Biotechnology Co., Ltd, Shanghai, China) as a protein standard. The tear samples were stored at −80 °C, and thawed only once before analysis.

### SDS–PAGE

SDS–PAGE was performed on a mini-vertical electrophoresis system (Bio-Rad Mini-PROTEAN^®^ 3 Cell; Bio-Rad Laboratories, Hercules, CA). Each tear sample with an equal amount of total proteins was separated on a 13% acrylamide resolving gel (0.1% SDS, 1.5 M Tris-HCl, pH 8.8) with a 5% acrylamide stacking gel (0.1% SDS, 0.5 M Tris-HCl, pH 6.8). Electrophoresis was performed in electrode buffer (0.1% SDS, 0.25 M glycine, 0.025 M Tris-HCl, pH 8.3) at 60 V for 10 min, and then switched to 120 V for 120 min. Each experiment was repeated thrice in different gels and running buffers.

### Two-dimensional gel electrophoresis (2-DE)

Tear proteins were precipitated by acetone according to the method of Green-Church [26]. Briefly, pre-chilled acetone was added to tear samples at fourfold volume of the sample to be precipitated. The tube was vortexed and incubated at −20 °C for 120 min. The precipitated proteins were pelleted by centrifuging at 4 °C for 10 min at 13,000× g. The acetone was discarded and the protein pellet in the tube was air dried.

2-DE was performed using reagents and instruments from GE Healthcare (GE Healthcare Bio-Sciences AB, Uppsala, Sweden), and according to our previously reported protocols [27,28], unless otherwise specified. First-dimensional isoelectric focusing (IEF) was performed using the Ettan IPGphor II unit. Protein samples (100 μg per gel) were diluted to 250 μl in a rehydration buffer (7 M urea, 2 M Thiourea, 2% CHAPS, 2.8 mg/ml dithiothreitol, 0.002% bromophenol blue, 0.5% pH 3–10 immobilized pH gradient buffer). IPG strips (13 cm, pH 3~10 linear gradient) were loaded with protein samples and rehydrated using the passive rehydration method for 1 h and the active rehydration method at 50 V for 11 h. Isoelectric focusing was run at 20 °C with the voltage settings of 500 V for 1 h (step and hold), 1,000 V for 1 h (gradient), 8,000 V for 3 h and 30 min (gradient), and lastly 8,000 V for 30 min (step and hold). The IEF strips were subjected to the standard equilibration steps before second-dimensional electrophoresis. The IEF strips were soaked for 15 min in the equilibration buffer (6 M urea, 50 mM pH 8.8 Tris-HCl, 2% SDS, 29.3% glycerol, 0.002% bromophenol blue, 1% DTT). They were then soaked for an additional 15 min in the same solution, except that 1% DTT was replaced with 2.5% idoacetaminde. The IEF strips were applied onto 13% SDS–PAGE. The second-dimensional SDS–PAGE was performed with vertical electrophoresis system (Amersham Pharmacia Biotech B, Uppsala, Sweden) at 60 V for 15min, 150 V for 2 h and 300 V for 3 h. Results of 2-DE were repeated thrice independently.

### Staining and image analysis

Gels were stained with hot Coommassie blue R-350 [29]. After finishing SDS–PAGE, gels were fixed in 40% (v/v) methanol and 10% (v/v) acetic acid for 1 h and then stained in a staining solution (0.025% Coommassie blue R-350 in 10% acetic acid) heated to 80~90 °C. The gels were destained in 10% acetic acid. All gel images were recorded immediately after destaining to minimize any possibility of fading. Images were acquired with the Kodak Image Station 4000MM (Kodak, Rochester, NY). For SDS–PAGE images, Quantity One (Bio-Rad Laboratories) was used to examine the lane profiles by calculating trace quantity and relative quantity of each band (the quantity of a particular band as measured by its intensity, expressed as a percentage of the total intensity of the lane) according to the Quantity One manual [30]. And 2-DE images were analyzed with Melanie Ver. 4.0 software (GeneBio, Geneva, Switzerland) by calculating volume intensity of each spot [28].

### Identification of tear proteins by mass spectrometry

The protein identification was performed using the methods reported before [27,31]. Briefly, the protein spots selected for identification were manually excised and subjected to in-gel digestion. Excised gel spots were destained at 37 °C with 25 mM ammonium bicarbonate/50% (v/v) acetonitrile (ACN) and then dehydrated with ACN. For digestion, the gel pieces were rehydrated in 25 mM ammonium bicarbonate solution containing 12.5 ng/μl trypsin (sequencing grade; Promega, Madison, WI) and incubated at 4 °C for 30 min. The supernatant was discarded; gels were incubated at 37 °C for 8 h in 25 mM ammonium bicarbonate. Finally, peptides were eluted and dissolved with 25 mM ammonium bicarbonate for MALDI-TOF/TOF-MS analysis. The matrix solution was prepared by dissolving R-Cyano-4-hydroxycinnamic acid (CHCA) in an ethanol/acetone mixture (2:1, v/v) to a final concentration of 1 μg/μl. Two μl sample followed by 0.1 μl matrix was applied to an Anchor Chip (Bruker Daltonics, Bremen, Germany). Crystallization occurred at room temperature. MALDI-TOF and MALDI-TOF/TOF spectra were acquired using an Ultraflex III TOF/TOF mass spectrometer (Bruker Daltonics, Bremen, Germany).

Due to the lack of genomic or proteomic database for camels, the protein annotations for *Camelus* in NCBInr database 201005 were loaded onto the Mascot search program. The peptide mass fingerprint (PMF) data combined with the corresponding MS/MS spectra data of the tryptic peptides derived from the gel spots were searched against the loaded protein annotations using the local Mascot search program, with the search parameters set as follows: Enzyme: Trypsin; Fixed modifications: Carbamidomethyl (C); Variable modifications: Oxidation (M); Mass values: Monoisotopic; Peptide Mass Tolerance: ±100 ppm; Fragment Mass Tolerance: ±0.5 Da; Max Missed Cleavages: 1. The identification of each spot was repeated three times.

### Western blotting

Rabbit anti-human vitelline membrane outer layer protein 1 (VMO1) polyclonal antibody reacting against to a region within amino acids 1 to 167 of human VMO1 (GeneTex Inc., San Antonio, TX) and rabbit anti-human lactoferrin (LF) antibody reacting against amino acids 650 to the COOH-terminus of human LF (Abcam Inc., Cambridge, UK) were used for western blotting to validate the 2-DE and mass spectrum results. Western blotting analysis was conducted according to specifications of the antibody manufacturer and our previous reports [27,28]. Briefly, equal amounts of total tear proteins were separated by 12% acrylamide SDS–PAGE, and then blotted onto the PVDF membrane using the mini trans-blot system (Bio-Rad). The membrane was blocked with 5% fat-free milk in TBST (Tris-buffered saline including 0.1% Tween) and incubated with the blocking solution containing 1:800 (VMO1 antibody) and 1:1,000 (LF antibody) dilution of the primary antibody at 4 °C overnight. The membrane was subsequently incubated with the blocking solution containing 1:4,000 dilution of goat-radish peroxidase-conjugated anti-rabbit IgG secondary antibody (Santa Cruz Biotechnology Inc., Santa Cruz, CA). Detection was performed with Phototope-HRP Western Blot Detection System (Cell Signaling Technologyy Inc., Danvers, MA). Western blotting was scanned and analysis with the Kodak Image Station 4000MM (Kodak). The result was repeated and confirmed in three independent tests.

### Statistics

The differences of each band densitometric values (trace quantity and relative quantity) between the tear samples collected in the summer and winter were assessed by independent sample *t*-test. The differences of VMO1 homolog and LF in western blotting between the tear proteins in the summer and winter group were evaluated by paired sample *t*-test. For all statistical tests, p<0.05 was considered significant.

## Results

### SDS–PAGE gel patterns of camel tears collected in the summer and winter

**Figure 1:**
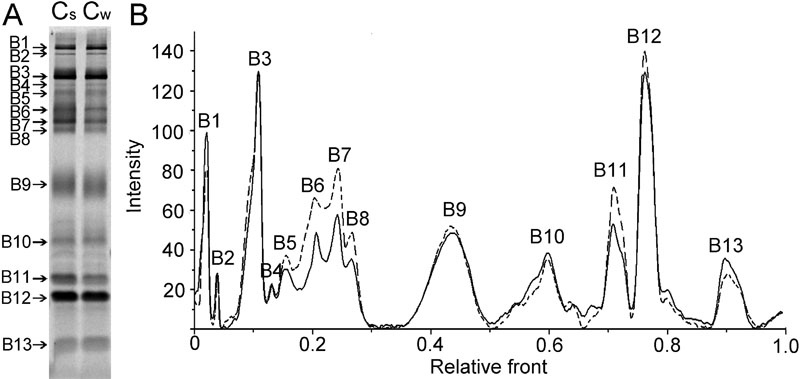
Comparison of SDS–PAGE gel patterns of proteins in camel tear fluids between summer and winter. **A**: Proteins of camel tears in the summer (lane Cs) and in the winter (lane Cw) were separated on a 13% gel with equal amount of total tear proteins in each sample. Thirteen well resolved bands are detected in both lanes. **B**: Graphic of lane comparison of camel tear proteins between the summer (dotted line) and the winter (solid line). B1, Band1; B2, Band2; B3, Band3; and so forth in **B** are correspondent with those in **A**.

To investigate the difference in protein composition of camel tears in different seasons, samples were initially analyzed by SDS–PAGE, loading equal amount of total tear proteins. The distribution of protein bands following SDS–PAGE was reproducible. About 13 well resolved bands were observed in both summer and winter camel tears (Figure 1A).

By band densitometry, the lanes graphic (Figure 1B) was showed and relative quantity of each band was calculated (Table 1). Comparisons of tears in two seasons were made and variable quantitative differences of several correspondent bands appeared to be present. The proportion of band 6, 7, and 11 in camel tears of the summer group (lane Cs) were 9.24±0.70%, 7.30±0.41% and 7.41±0.28%, significantly higher than those of the winter group (lane Cw) as 6.15 ±0.51% (p=0.004), 5.99 ±0.24% (p=0.009) and 5.64 ±0.72% (p=0.016), respectively, while the rate of band 13 (4.43 ±0.23%) in the summer group showed less than the one in the winter group (6.01 ±0.88%) significantly (p=0.040). Because differences in tears from two seasons were detected, we attempted to identify proteins contained in each tear using 2-DE and MALDI-TOF/TOF-MS.

**Table 1:** The comparison of relative quantity^#^ of protein bands of camel tears in SDS–PAGE gels between summer and winter.

**Band number**	**Cs (%)**	**Cw (%)**	**p value**
B1	5.76±2.27	6.95±2.22	0.554
B2	1.63±0.27	2.13±1.10	0.490
B3	12.86±0.71	12.54±0.31	0.513
B4	1.84±0.24	2.06±0.53	0.550
B5	4.64±1.08	4.49±1.20	0.881
B6	9.24±0.70	6.15±0.51	0.004**
B7	7.30±0.41	5.99±0.24	0.009**
B8	3.81±0.16	3.44±0.38	0.188
B9	12.65±0.75	13.77±0.54	0.102
B10	3.92±1.03	4.25±0.72	0.669
B11	7.41±0.28	5.64±0.72	0.016*
B12	12.82±0.81	13.02±1.27	0.829
B13	4.43±0.23	6.01±0.88	0.040*

^#^: Relative quantity of each band: the quantity of a particular band as measured by its intensity, expressed as a percentage of the total intensity of the lane. Values are presented as mean±standard deviation (%). p-values are determined by independent sample *t*-test. p<0.05 is considered significant. (*: p<0.05; **: p<0.01) Band number is correspondent with ones in Figure 1A,B.

### 2-DE proteome profiles of camel tears in two seasons and protein identification

**Figure 2:**
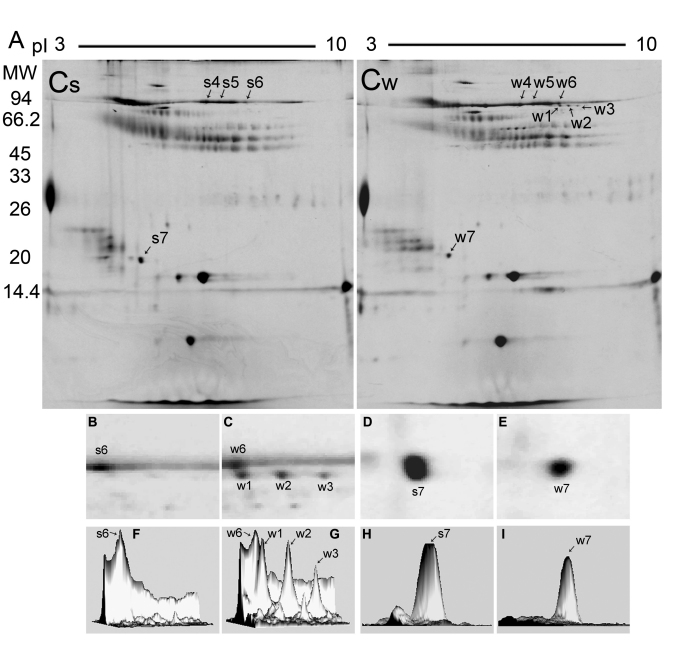
Comparison of 2-DE Coomassie-stained protein profiles and differential expression spots of camel tears between summer and winter. **A**: Tear proteins (100 μg) in the summer (Cs) and in the winter (Cw) were separated on first-dimensional pH 3–10 linear IPG gels (13 cm) and second-dimensional 13% vertical slab gels. The relative MW is given on the left, while the pI is given at the top of the figure. The spots marked by arrows and numbers were cut and digested, and then identified using MALDI-TOF/TOF-MS. B-I: Protein spots w1, w2, w3 and s7, and w7 with different volume intensities are displayed in the enlarged spot views of 2-DE images (**B**-**E**) and as three-dimensional images obtained by Melanie 4.0 software (**F**-**I**). Spots w1, w2, w3, w4, w5, w6 and s4, s5, and s6 were identified as LF and spots s7 and w7 were characterized as VMO1 homolog. **B**, **D**, **F**, **H**: The summer group (Cs); **C**, **E**, **G**, **I**: The winter group (Cw).

In this study, we presented the comparative report of the 2-DE protein reference maps of camel tears collected in the summer and winter (Figure 2A), as a basis for subsequent differential expression proteomic studies on tears in various seasons. Comparison of the spots in 2-DE gels in the summer and winter groups through Melanie ver. 4.0 software revealed that there were differential expressions of proteins in spots w1, w2, w3 and s7, w7 (Figure 2B-I). So these spot gels were excised and identified by in-gel digestion and MALDI-TOF/TOF-MS analysis.

**Figure 3:**
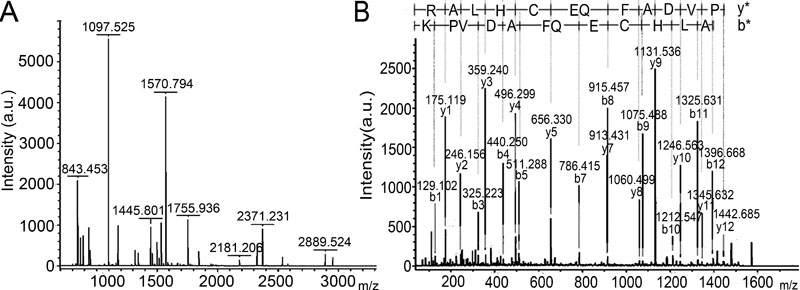
The MALDI-TOF/TOF mass spectrum analysis of spot w6 in Figure 2 indentified as LF (*Camelus dromedarius*, gi|5777368). **A**: The PMF signals. **B**: The MS/MS spectrum of parent ion 1570.794 for the sequence KPVDAFQECHLAR calculated by b ions (b*) and y ions (y*).

Spots w1, w2, w3 of about 78 kDa were detected in the winter group but failed to be detected in the related area of the summer group. Then these spots were all identified as lactoferrin (*Camelus dromedarius*; gi|5777368), identical to the identification results of spots s4, s5, s6 in the summer group and spots w4, w5, w6 in the winter group with 79 kDa in the 2-DE gels (Figure 2). Figure 3 shows the representative results of the PMF of LF (spot w6) combined with the MS/MS spectrum of one of parent ions (1570.794) for the sequence KPVDAFQECHLAR.

The volume intensity of spot s7 in summer group showed higher than the one of spot w7 in winter group (p=0.014). According to our analysis by MALDI-TOF/TOF-MS with a combined strategy of de novo sequencing and BLAST homology searching (data not shown), it was characterized as VMO1 homolog which has not been reported in the tear fluids in others’ studies.

### Differentially expressed proteins confirmed by western blotting

**Figure 4:**
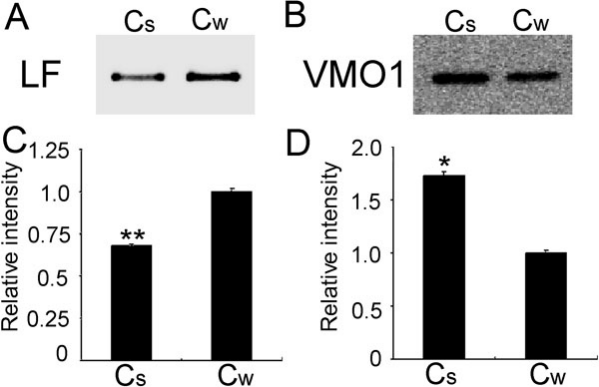
Western blot analysis of decreasing expression of LF and increasing expression of VMO1 homolog in camel tears in the summer compared to the winter. **A**, **B**: Comparison of expression of LF (**A**) and VMO1 homolog (**B**) between the summer group (Cs) and the winter group (Cw) by western blotting. **C**, **D**: Relative quantitative analysis of each corresponding band of LF (**C**) and VMO1 homolog (**D**) in two groups, based on the volume intensity of the band in Cw as 1.0. The paired student’s *t* test was performed and showed a significant difference (*p=0.042, **p=0.002) between two groups.

Differentially expressed LF and VMO1 homolog in tear fluids between the summer and winter groups were further confirmed by western blotting (Figure 4). The approximately 79 kDa of LF and 21 kDa of VMO1 homolog were both detected in the tears of the summer and winter groups. There was a significant seasonal variation that the expression of LF (p=0.002, Figure 4A,C) in the summer group was reduced but the expression of VMO1 homolog increased (p=0.042, Figure 4B,D) compared to the winter group.

## Discussion

Seasonal shift between the cold weather and the hot weather would result in different environmental conditions, e.g., temperature, relative humidity and air flow. Camels survive in the markedly harsh living environment with the extremely heat and cold weather. Evaluation of the seasonal variation of camel tear proteins may provide us a clue to verify the role of tear proteins in the maintenances of the ocular surface, such as stabilizing the tear film, under environmental risk factors. In this study, camel tears were collected near Riyadh (24°38′N, 46°43′E), Saudi Arabia where the overall climate is arid and has many dust storms. The average high temperature from June to July is around 45 °C and the average low temperature from December to January is around 7.0 °C. The average relative humidity is from 10% in June – July to 47% in December – January. Our results have demonstrated that there was an obvious seasonal variation on electrophoresis patterns, with a decrease in LF and an increase in VMO1 homolog in the hot season compared to the cold season. These two abundant proteins in camel tears have been detected to be varied in the concentrations in two seasons. Meanwhile, it is likely that other tear proteins including less abundant proteins or small molecular weight proteins may also have various expression profiles in different seasons.

The seasonal variation of the composition of proteins in the tear fluids maybe reflects a mechanism of animals to keep ocular surface environment in balance under harsh circumstance. The influence of seasonal factors on the tear secretion has been shown in Harderian gland (HG), which is an orbital gland found in many tetrapod species that possess a nictating membrane and is presumed to lubricate the eye in the absence of a lachrymal gland [32]. The HG of frog (*Rana esculenta*) was reported to have seasonal secretory activity changes which were consistent with the changes of environmental temperature and correlated well with alteration of kinase expressions [32,33]. In our study, seasonal changes of tear protein composition were, at least in part, associated with the modification of seasonal secretory activity of ocular glands and other secretory tissues, as one of physiologic features in camel eyes for the adaptation to the changes of environment conditions in different weathers. The opposite quantitative changes of LF and VMO1 homolog was able to exclude the reason contributed only by evaporation of tear film or alteration of tear volume which would lead to consistent changes in the amount of these two proteins.

Tear LF is a member of a transferrin family, a metal binding glycoprotein with an important role in antimicrobial, anti-inflammatory and oxygen free radical and hydroxyl scavenging activities [3,34], accounting for approximately 25% of the total tear protein in human being [35]. Besides the lacrimal gland is the major source of tear LF [36], conjunctival and corneal epithelial cells have been revealed to produce detectable amounts of LF [34] and the meibomian gland might also serve as a source of LF in tear film [37]. Diurnal variation in LF levels is found between open-eye (waking) and closed-eye (sleeping) tear samples [3,38,39]. LF, lysozyme and lipocalin have been demonstrated to account for ~85%–88% of the total protein in basal-type and flex-type open eye tear samples, but decreased to less than ~30% of the total protein in closed eye tear samples [38]. No change has been reported in LF concentration between reflex tears and (basal) open-eye tears (~30% relative to the total protein) but a decrease to 10% of the total protein in the closed eye [3]. Moreover, Willcox et al. [39] found the function of inhibiting complement of LF in closed-eye tears was reduced. Our study first revealed that there was seasonal variation of LF levels in camel tears. Decreased LF in the summer was inferred to be probably the result of its reduced synthesis or secretion for different neural/hormonal responses to stresses of the environment (temperature, humidity, or pathogenic) [40].

Evidences have been presented to show that several apparent molecular weight (MW) forms of LF from different secretions and tissues were separated by SDS–PAGE [41-44]. The analysis of human tears under reducing conditions indicated that MWs of LF varied between 78 kDa for the major band and 83 kDa for the minor band in SDS–PAGE gels [41]. It was suggested that the glycosylated nature of protein may be one of the causes of the MW forms of LF [41-44]. In our study, varied MW forms of LF (spots w1, w2, w3, w4, w5, w6, s4, s5, and s6) were also detected in the 2-DE gels. However, in the analysis by MALDI-TOF/TOF-MS, we didn’t find any significant differences of the PMFs and MS/MS spectrum between lower MW spots (w1, w2, and w3) and higher MW spots (w4, w5, w6, s4, s5, and s6). Therefore, according to the studies previously reported [41-44], the phenomenon may be also likely due to posttranslational modifications (e.g., glycosylation).

VMO1 homolog is being reported for the first time in camel tears and has not been found in human tear proteome profiles reported before [45,46]. No previous studies have reported similar findings. Chicken VMO1, 183 amino acids, a secreted protein, was first characterized in the outer layer of the vitelline membrane of poultry eggs together with lysozyme, VMO2 and ovomucin by Back et al. [47] in 1982. Interestingly, by comparing the components of various tissues, we found that the composition of abundant proteins in human tear fluids was similar to those in chicken egg white [48] and egg vitelline membrane [49], which mainly comprise of VMO1, ovalbumin, lysozyme C and ovotransferrin. Shimizu et al. have analyzed the crystal structure of VMO1 and spectulated that VMO1 might have an enzymatic activity related to saccharides [50]. Though the origin and the exact function of VMO1 in camel tears remain obscure, its existence in camel tears and increasing level in the summer provide us a clue of its important role in ocular surface maintenance under harsh circumstance. Further studies need to be performed to verify its role in tear fluids and the relationships with the abundant protein components of tear fluids including LF.

Our current results would contribute to studies on the ocular surface of human and animal eyes. Seasonal alterations of tear proteins in camels indicate that environmental risk factors may also exert the influence on human tear proteins, which function in the maintenance of ocular surface. In previous reports, tear LF assays have been used as a predictor of tear film stability or tear volume change in clinical practices since decreases in LF concentration are correlated with decreases in tear production from the lacrimal gland in dry eyes [51-53]. Additionally, the alteration of tear film evaporative rate and the dysfunction of lipid layer were demonstrated to be involved in environment related ocular discomforts including dry eye [10,15]. However, no evidence has proved that the variation of protein expression (e.g., LF) in tear fluids of humans or animals was associated with environmental changes (low relative humidity and high temperature etc.). The present data offers us a hint of potential molecules in the pathogenesis of human ocular surface disorders induced by the environmental factors. Also, further confirmation of the function of VMO1 in camel tear fluids may promote a novel try in the therapy of ocular surface diseases. Therefore, it’s valuable to explore the role of tear proteins (e.g., LF and VMO1) in the physiologic and pathophysiological process of ocular surface under environment risk factors. In addition, the finding would also suggest that the annual time and environment conditions should be taken into consideration during the evaluation of tear proteome.

In conclusion, our study indicated there was seasonal variation of protein composition including LF and VMO1 homolog in camel tear fluids. Although further studies are required to examine the mechanism why tear proteins varied seasonally, the results will be helpful in expanding the knowledge of physiologic characteristics of tear fluids, as a basis for advanced investigation into seasonal effect on human tear proteomic changes and exploration of potential application for the diagnosis and treatment of ocular surface disorders.
